# Flexible Magnetic Sensors

**DOI:** 10.3390/s23084083

**Published:** 2023-04-18

**Authors:** Lili Pan, Yali Xie, Huali Yang, Mengchao Li, Xilai Bao, Jie Shang, Run-Wei Li

**Affiliations:** 1 CAS Key Laboratory of Magnetic Materials and Devices, Ningbo Institute of Materials Technology and Engineering, Chinese Academy of Sciences, Ningbo 315201, China; panlili@nimte.ac.cn (L.P.); limengchao@nimte.ac.cn (M.L.); baoxilai@nimte.ac.cn (X.B.); runweili@nimte.ac.cn (R.-W.L.); 2 Zhejiang Province Key Laboratory of Magnetic Materials and Application Technology, Ningbo Institute of Materials Technology and Engineering, Chinese Academy of Sciences, Ningbo 315201, China; 3 School of Future Technology, University of Chinese Academy of Sciences, Beijing 100049, China

**Keywords:** flexible magnetic sensors, flexible electronics, shapeable magnetoelectronics, Hall sensors, magnetoresistance (MR) sensors, magnetoimpedance (MI) sensors, magnetoelectric (ME) sensors

## Abstract

With the merits of high sensitivity, high stability, high flexibility, low cost, and simple manufacturing, flexible magnetic field sensors have potential applications in various fields such as geomagnetosensitive E-Skins, magnetoelectric compass, and non-contact interactive platforms. Based on the principles of various magnetic field sensors, this paper introduces the research progress of flexible magnetic field sensors, including the preparation, performance, related applications, etc. In addition, the prospects of flexible magnetic field sensors and their challenges are presented.

## 1. Introduction

With the rapid development of the Internet of Things, wearable and implantable technology, flexible electronics have attracted much attention [1,2]. Flexible electronics enable electronic devices to be arbitrarily reshaped as required after manufacturing. Electronic components, such as optoelectronic components, magnetic electronic components, and energy storage components, have the characteristics of scalability, biocompatibility, non-damage, lightweight, and miniaturization, and they can be applied to various complex surfaces, including human skins [3]. Because of these unique advantages, flexible electronics have been widely used in many fields such as intelligent robots [4], health care [5], electronic skins (E-Skins) [6,7], and human-computer interactions [8], and the study of flexible electronics has gradually become an important research direction worldwide.

Among them, magnetic electronic components have a wide range of applications in the fields of driving, energy conversion, information sensing, processing, and storage [3,9,10]. The integrated flexible magnetic sensor can convert the change of magnetic performance of magnetic electronic components caused by external factors into electrical signals and can detect the presence, strength, direction, and the change of magnetic field [11]. Because of the three-dimensional universality of magnetic fields, they can be used in non-contact detection platforms and unlock more human-computer interaction scenes for intelligent systems, eliminating the camera and other devices required by optical systems [1,6].

With the change and development of application requirements, the traditional magnetic sensor manufactured on rigid silicon substrate has gradually failed to meet the requirements, so the concept of flexible magnetic sensor was put forward. So far, research on flexible magnetic sensors is still in its infancy [6,11,12,13,14]. Depositing magnetic multilayers on flexible organic substrates without sacrificing performance remains challenging. Firstly, the surface roughness of the flexible substrate will affect the performance of the magnetic film. Secondly, in thermal, chemical, or micromachining processes, flexible substrates are more vulnerable to damage than rigid substrates.

This review summarizes the latest development of flexible magnetic sensors. We summarize the working principle, application range, and characteristics of flexible magnetic sensors made by different sensing principles. The first part mainly introduces the research progress of the flexible magnetic field sensors, with special attention to their preparation methods and performances. The second part provides examples of applications such as geomagnetosensitive E-skins, magnetoelectric compass, and non-contact interactive platform, and so on. In the last part, prospects and challenges of flexible magnetic field sensors are presented.

## 2. Flexible Magnetic Sensor Devices

### 2.1. Flexible Hall Sensor

**Figure 1:**
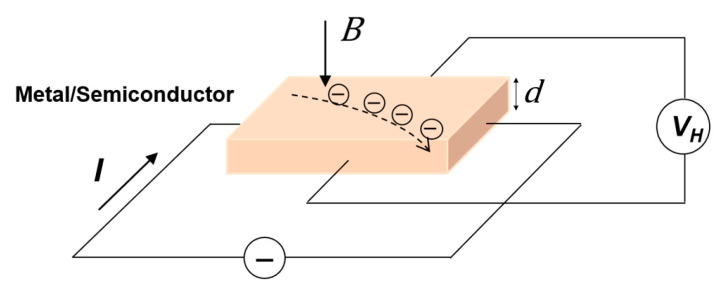
Working principles of Hall sensor.

Hall effect was observed by Edwin H. Hall in 1879 [15]. As shown in Figure 1, when the current passes through semiconductors or very thin conductive plates perpendicular to the external magnetic field, the charge carrier is deflected, and an electric field is generated perpendicular to the current and the magnetic field. The Hall voltage is expressed as

(1) $$V_{H}=\frac{IBR_{H}}{d}$$

where *I* is the current, B the magnetic field, $R_{H}$ the Hall resistance, and d is length.

Hall sensor uses Hall effect as the core principle to convert the measured magnetic field into electromotive force, which has a broad application range [11]. With the change and development of application requirements, the traditional rigid semiconductor sensor with a thickness of approximately 400 μm cannot meet the purpose [16], so flexible Hall sensor has been developed, which is stable and wearable.

**Figure 2:**
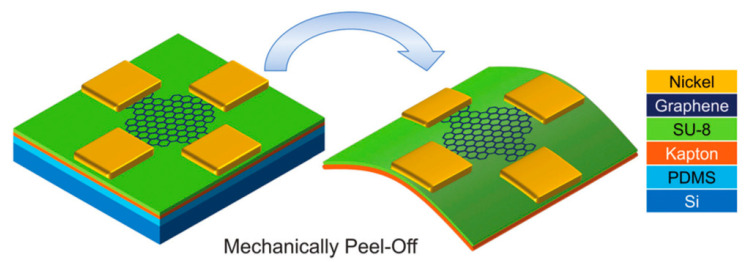
The device architecture of the flexible Hall sensor is based on graphene. Adapted with permission from Ref. [17]. Copyright 2016, copyright Royal Society of Chemistry.

Graphene is the two-dimensional carbon allotrope, and carrier mobility μ and lamellar carrier density n are two important parameters to measure the sensitivity of graphene-based sensor, which can be expressed as

(2) $$S_{V}=\frac{1}{V}\left|\frac{\partial V_{H}}{\partial B}\right|=\frac{\mu W}{L}$$

(3) $$S_{I}=\frac{1}{I_{C}}\left|\frac{\partial V_{H}}{\partial B}\right|=\frac{L}{ne}$$

where *W* and L are the width and length of the Hall sensor device, and *e* is the elementary charge. It offers high carrier mobility, low sheet carrier density, and excellent flexibility for both rigid and flexible substrates, which can be considered as the perfect material for Hall sensors. In the work of [17], a flexible Hall sensor based on graphene was fabricated (Figure 2), which shows the sensitivity up to 79 V/AT, comparable to rigid silicon-based Hall sensors and outperforming other flexible Hall sensors.

A key component of navigation and position tracking devices for in vivo evaluation of mechanical motion is Hall sensor. Melzer et al. fabricated flexible bismuth Hall sensors on polyether ether ketone (PEEK) and polyimide (PI) foils that can be in close contact with the human body to enable an interactive pointing device that visualizes the position of the finger relative to the magnetic field [18]. And then Monch et al. improved the reliability and made it suitable for the measurement of time-varying magnetic fields, enabling realization of the flux-based control scheme and integrating two sensors of curved structure into the flux density control loop of a single degree of freedom spherical maglev device successfully [16].

A flexible Hall sensor with its ultra-thin, reliable, flexible characteristics is used to achieve packaging in some engineering applications, such as active magnetic bearings (AMBs), of which the stiffness and precision becomes higher as the gap decreases. It fits the AMB perfectly to make the gap as small as possible, resulting in improved operation by measuring the magnetic flux inside the air gap in between the curved stator and rotor components [19]. Arkadiusz et al. integrated it into the AMB air gap with signal amplifiers, with sensitivity comparable to rigid sensors, realizing the flux-measurement system for the 1-degree of freedom (DoF) AMB system [20]. The control method for AMBs requires real-time data of the air gap flux density, and the sensor must be placed in the air gap of the AMB to measure the air gap flux density. PI film can be used as the sensor intermediary layer, eliminating the use of cylindrical convex process, further reducing the thickness of the sensor system, and optimizing the packaging process of polyimide-based Hall sensors for AMBs [21]. 

The use of polymeric foils and current spinning technique compensate for the effects caused by mechanical stress and possible fabrication imperfections, meeting the needs of wireless communication [6]. In 2016, Hadi et al. successfully fabricated a magnetic sensor chip, integrating four different vertical Hall sensors on PVC and FPC using 0.18 μm CMOS technology, which can maintain high sensitivity of sensors in a large magnetic field range [22]. 

Flexible Hall sensors, featuring high sensitivity, large detection range, and simple manufacturing, may be used in soft robots, interactive devices in virtual and augmented reality, and point-of-care platforms for detecting magnetic objects.

### 2.2. Flexible AMR Sensor

**Figure 3:**
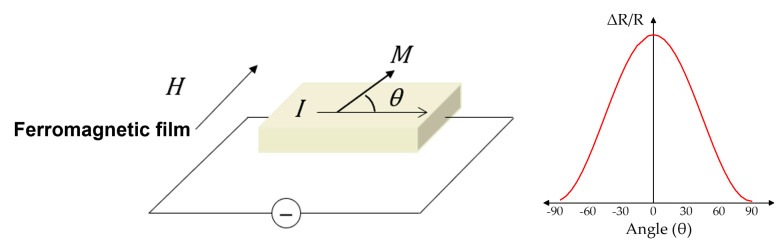
Working principles of AMR sensor.

The anisotropic magnetoresistance (AMR) effect, discovered in 1857 by William Thomson [23], is a phenomenon in which the resistivity of a ferromagnetic material varies depending on the angle between the magnetization and the direction of the current in Figure 3. When the direction of the current passing through the material is perpendicular to the direction of the applied magnetic field (90°), the resistance is small, and when the two directions are parallel (0° or 180°), the resistance is large. Unlike other MR effects, the AMR effect is based on anisotropic scattering of conducting electrons with uncompensated spin.

The signal generated by the computer hard disk read head is larger than that generated by the traditional induction coil type read head, so AMR sensors have been widely used in the hard disk read head and magnetic devices, which are advantageous in terms of their small volume, simple process, low cost, low impedance, and high reliability. They are also immune to harsh environment (light, dust, liquids, etc.) based on the AMR effect.

Early on, to create “genetically intelligent” (gentelligent) machine tools or components, various microsensors have been developed to collect process data during the production process, even throughout the life cycle [24,25]. Traditional MEMS systems manufactured on Si substrates have gradually failed to meet the requirements, so a concept for the fabrication of modular micro sensors on a flexible substrate has been proposed [26].

In 2011, an AMR sensor was successfully fabricated on SU-8™ layer with Si as substrate and Cr and SiO_2_ as sacrificial layers, which is the fabrication of the first modular microsensor and is also the first to be flexible. Since then, the importance of sacrificial layer research has been proved [26]. In the work of [27], the AMR microsensor was fabricated on a flexible polymer foil with a thickness of 7 μm, released from the Si frame by die cutting with a stamping tool, which makes wafer thinning and wafer separation processes for this microsensor unnecessary.

In addition to the sacrificial layer method, there were researches on the growth of AMR sensors on polymer films such as polyethylene terephthalate (PET) [28], Kapton [29,30], polydimethylsiloxane (PDMS) [31]. In these fabrication processes, the most important factor affecting the performance is the interaction between the polymer film and the functional layer of the sensor, such as internal stress and roughness [30].

A self-biased AMR sensor, which is based on the Wheatstone bridge and barber pole structures, on the PR buffered flexible PET foils was fabricated. The current flow can be rotated by an angle of 45° or 135° with respect to the magnetic easy axis [32]. In 2016, Wang et al. fabricated a self-biased AMR sensor with excellent stability and a sensitivity of 4200%/T, which is close to that of the AMR sensors on rigid Si substrates and GMR sensors on flexible or rigid substrates [28].

**Figure 4:**
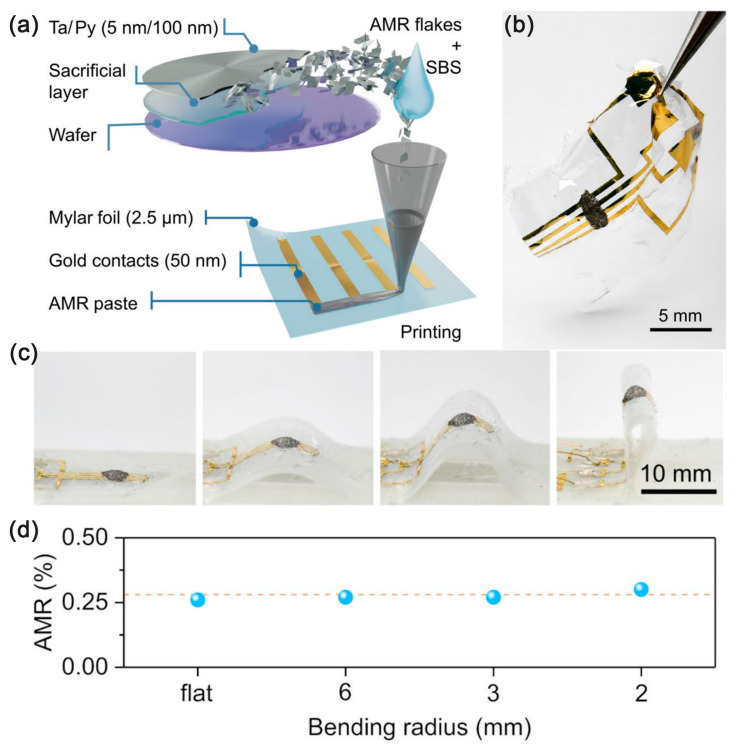
(**a**) Schematics on the fabrication process of printed AMR sensors; (**b**) Physical picture of the printed AMR sensor; (**c**) Photographs revealing the bending state of the sensor; (**d**) the respective AMR response measured at ±400 mT. Adapted with permission from Ref. [33] and licensed under CC BY 4.0. Copyright 2021, copyright Springer Nature Limited.

To improve the deformability of the sensor, a mixture of AMR flakes and an elastomer have been printed on 2.5 μm polyester foil as a paste (Figure 4), which can be folded with a bending radius of 20 μm and has no loss of performance stability [33].

**Figure 5:**
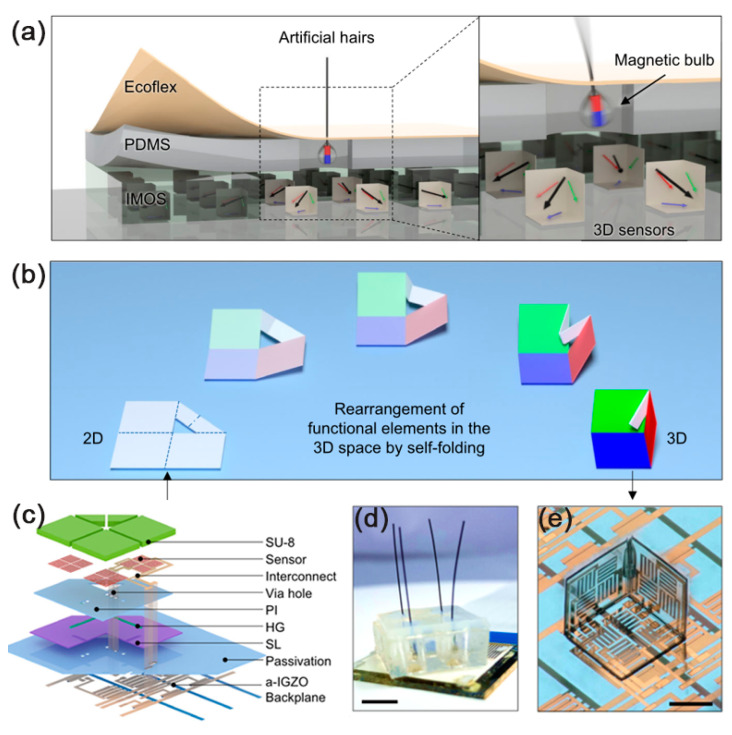
(**a**) Vision of the active matrix integrated micro-origami magnetic sensor for E-skin application; (**b**) Deterministic self-folding process; (**c**) Layout design of the self-foldable platform; (**d**) Photograph of the magnetic hair embedded e-skin system. Scale bar, 5 mm; (**e**) One pixel in the 3D sensor. Scale bar, 200 µm. Adapted with permission from Ref. [34] and licensed under CC BY 4.0. Copyright 2022, copyright Springer Nature Limited.

With the development of active matrix magnetic sensors, a high density integrated magnetic sensor with three-dimensional (3D) magnetic vector field sensing capability has been fabricated by combining the AMR effect with them (Figure 5), which is composed of a group of self-assembled miniature origami cubic structures. Real-time multidirectional tactile perception can be achieved by integrating 3D magnetic sensors into E-skin embedded in magnetic hairs [34]. 

In 2018, a flexible and mechanically robust AMR sensor-based E-skin compass system was developed, which combines magnetic field detection capabilities and mechanical endurance with skin electronics, driven by the Earth’s magnetic field and can be bent up to 150 μm with stability [31].

**Figure 6:**
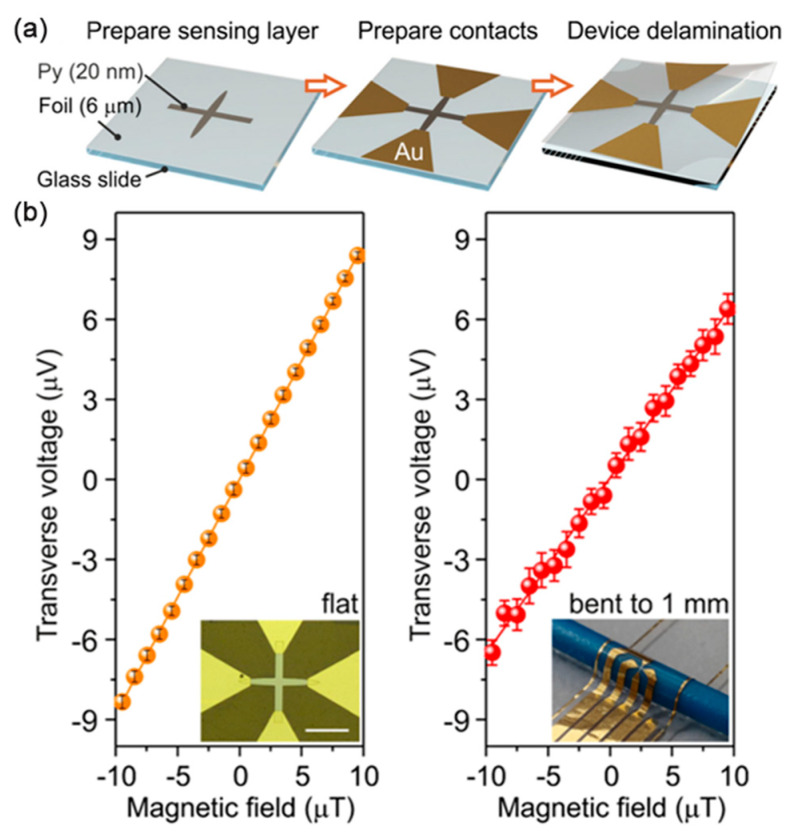
(**a**) Schematics on the fabrication process of flexible PHE sensor; (**b**) Magnetoelectrical characterization of flexible PHE sensors in flat state and bent to 1 mm. Adapted with permission from Ref. [36] and licensed under CC BY 4.0. Copyright 2019, copyright Springer Nature Limited.

To improve the detection limits of high compliance and linear magnetic field sensors, more and more attention has been paid to planar hall effect (PHE). PHE is actually a measurement of the transverse magnetoresistivity due to spin-orbit coupling, which shares the same physical origin as AMR (which records the longitudinal magnetoresistance) but has higher resolution in the low frequency range [35]. Field sensors based on the PHE are good at sensing weak fields below the geomagnetic field because it is essentially linear around the zero field and exhibits high sensitivity. It has advantages such as high signal-to-noise ratio, small bias voltage, and small-field linear response, but there are many challenges in successfully manufacturing it on formable surfaces, such as substrate preparation and micromachining difficulties. As shown in Figure 6, Pablo et al. prepared a flexible PHE sensor that reduced the minimum detection limit of magnetic fields to 20 nT, showing high sensitivity of 0.86 V/T. Built on a mechanically imperceptible polymer foil with a thickness of only 6 μm, the sensor consisting of a magnetic layer (NiFe 20 nm) and a contact layer can be bent to a radius of 1 mm without any degradation of resistance [36]. Burak et al. proposed a formable planar Hall sensor with Hall rod geometry with bilayer structure of NiFe (10 nm)/IrMn (8 nm), which was first prepared on a flexible Kapton/PDMS substrate. The transverse voltage of the Hall-bar structure is PHE signal, and it exhibits a stable sensitivity of 7.4 V/AT. In addition, the typical AMR signal under concave and convex bending conditions are sensitive [37].

Emulating the behavior of PHE sensors, planar Hall resistance (PHR) sensors has the Whetstone Bridge with AMR structure, which has the advantages of high signal-to-noise ratio, excellent linear response to low filed, and low power consumption [38]. Kim et al. reported the effect of bending on the performance of PHR sensors grown on PDMS and parylene C polymeric substrates, and the sensor thin film on the substrates still conducts electricity because the cracks are very short [39].

Flexible AMR sensors, with high sensitivity, excellent flexibility, and stability, provide a more flexible platform for traditional magnetic pattern recognition, on which wearable electronic devices for navigation, medical diagnosis, and health monitoring can be realized. 

### 2.3. Flexible GMR Sensor

The giant magnetoresistance (GMR) effect, discovered independently by Albert Fert and Peter Grunberg, involves the use of alternating layers of magnetic and non-magnetic materials to produce large changes in electrical resistance in response to a magnetic field. Many studies have revealed that the GMR effect exists in the magnetic multilayers alternately grown in various ferromagnetic layers (such as Fe, Co, Ni, and their alloys) and nonmagnetic layers (such as Cu and Cr) [40,41,42]. The main source is from the related scattering of spin electrons as carriers in magnetic multilayer films, which was initially found in the alternating deposition of magnetic and nonmagnetic metal film stacks by molecular beam epitaxy [40].

**Figure 7:**
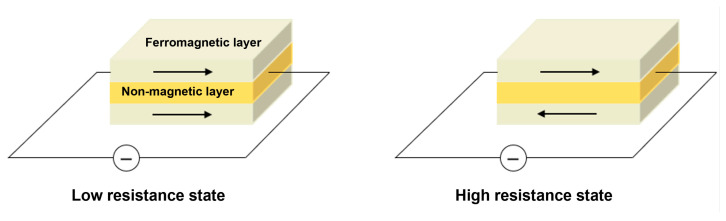
Working principles of GMR sensor.

Spin valve structure based on the GMR effect is composed of antiferromagnetic pinning layer, magnetic pinned layer, nonmagnetic metallic separation layer, and free magnetic layer [43,44]. With the development, the structure has evolved from a simple four-layer film structure to a multilayer film structure with additional buffer layer, composite free layer and pinning layer, and even a double-layer spin-valve structure, which makes the magnetic nano-film have better comprehensive properties. Their core principles are both based on the scattering of spin electrons. As shown in Figure 7, when the magnetization direction of the free layer and the pinned layer is antiparallel, the structure is in a high resistance state. When the magnetization direction is parallel, the multilayered structure is in a low resistance state. It was very suitable for magnetic sensors, which has a relatively large magnetoresistance ratio, low saturation field, and high magnetic field sensitivity.

In 1992, Parkin et al. sputtered and deposited GMR multilayers on Kapton organic film for the first time and obtained almost the same magnetic properties as GMR multilayers prepared on rigid silicon wafer under the same conditions [45]. Later, they sputtered a better exchange bias sandwich structure on various organic polymer films and obtained good GMR performance [46]. From then on, they combined flexible electronic technology with magnetic sensors, opening a new development process of flexible GMR sensors. However, Parkin’s method still does not overcome the shortcomings of sputtering, and the organic substrate used in this method must be very stable at high temperature.

Because the popular application of magnetism at that time was magnetic memory, the application and development of magnetic sensors were limited to the reading of auxiliary magnetic signals and did not receive much attention. In 2002, Yan et al. used the electrochemical synthesis method to electrodeposit Co/Cu multilayer on PPy polymer film and found that the interfacial roughness of polymer film was the key factor affecting the magnetoelectric properties of GMR multilayer film, and only 1 nm roughness would significantly reduce GMR properties [47]. To optimize GMR performance, it is necessary to reduce the impact of roughness. This can be achieved by coating an additional buffer layer on the polymer film [48] or depositing more periodic GMR multilayer films to increase the number of antiferromagnetic coupling layers. Both of these methods can achieve a value similar to the roughness of rigid silicon wafers and can enhance the performance of GMR multilayer films deposited on flexible substrates to 115–200% [49]. Another option is to grow a magnetic multilayer film on a rigid silicon wafer and then thin it to make the entire device flexible, such as a polymer film [50].

The performance of GMR films prepared on flexible substrates vary with the change of stress and strain. In 2006, German Siemens deposited the GMR film on PI, photolithographed and patterned, and carried out micro-nano processing, so that the material can withstand 2.5% tensile strain without changing its performance, providing a new direction for the miniaturization development of flexible GMR magnetic sensors [51]. In 2009, Anwarzai et al. combined magnetostrictive material (Co/Au/Co) with flexible PI film and proposed the possibility of applying this structure to flexible strain sensors [52]. In the work of Liu et al., they used FeGa alloy with magnetostrictive property to improve the strain sensitivity of the film and used composite free layer to avoid the reduction of GMR ratio, thus preparing a strain sensitive spin valve, which can be used for stress sensing [53]. Flexible GMR sensors are stress-sensitive because of the magnetostrictive effect of the ferromagnetic material, that is, the magnetic anisotropy can increase or decrease under stress depending on the magnetostrictive properties [54,55]. This property is suitable for use as a stress sensor, which can detect both the magnitude and the direction of stress [56,57]. At the same time, non-contact stress sensing can be achieved by adding permanent magnetic materials, such as flexible cilia [58] or films [59] doped with magnetic particles. The magnetic properties of the magnetic sensor can also be regulated by controlling the applied stress [54].

To endow the flexible GMR sensor with stretchability, Melzer et al. directly sputtered and deposited the Co/Cu multilayer film on the elastic PDMS film in 2011. The performance is equivalent to that on the silicon wafer and will not change significantly within 4.5% tensile strain [60]. In 2012, they demonstrated the application of flexible GMR sensor based on PDMS to the flow detection of magnetic particles in microfluids [61]. They were the first to successfully fabricate a flexible microfluidic analysis device based on a GMR magnetic sensor in 2014, which can detect magnetic objects encapsulated in lotion droplets [62]. During the preparation of PDMS substrate, the transverse thermal shrinkage strain of PDMS is restrained by the rigid silicon wafer attached to it, and the thermal stress will be effectively stored in PDMS to make the PDMS film obtain pre-strain [63]. Because the thermal expansion coefficients of PDMS film and metal film are different, after sputtering deposition, the film cools down from high temperature and releases thermal stress after stripping, resulting in random wrinkles and periodic cracks [64]. This fold obtained by thermally induced pre-strain can increase the tensile property of the flexible GMR magnetic sensor from 2% to 4.5%, while the mechanically induced pre-strain can increase the tensile property of the GMR magnetic sensor from 4% to 30% [65], and the polymer film with higher elasticity than PDMS can even reach 270% of the tensile property [66].

With the appearance of the stretchable GMR sensor [65,66,67,68,69], it was found that in addition to the interface roughness of the film substrate that will affect the device performance, the cracks caused by the direct deposition of the GMR multilayer films on the high elastic polymer film easily led to the reduction of GMR performance.

**Figure 8:**
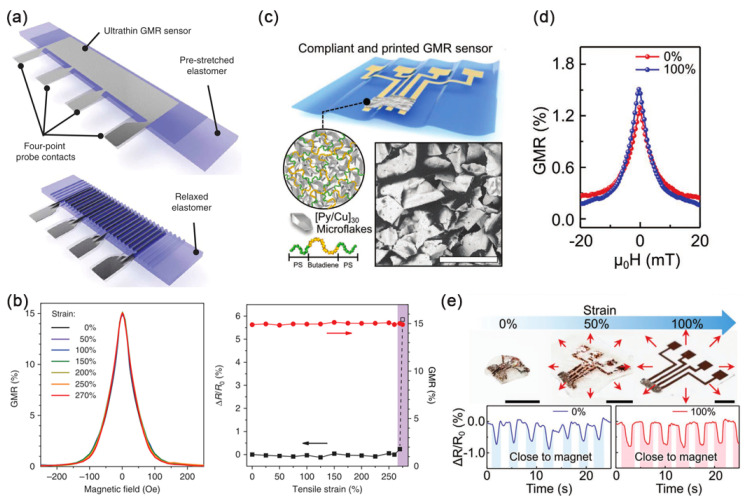
(**a**) Schematics of stretchable GMR sensors; (**b**) Stretching experiments results of the stretchable GMR sensor. Adapted with permission from Ref. [66] and licensed under CC BY 4.0. Copyright 2015, copyright Springer Nature Limited. (**c**) Schematics of printed GMR sensors; (**d**) GMR performance of the printed sensor in the relaxed and stretched state; (**e**) Photograph of a printed GMR sensor from 100% of stretching to 0% of relaxed state. Adapted with permission from Ref. [72] and licensed under CC BY 4.0. Copyright 2021, copyright John Wiley & Sons, Inc.

Researchers proposed two solutions (Figure 8): one is to avoid direct deposition on the polymer film by surface wrinkling, transferring, or rigid islands [70], and the other is to peel and grind all the magnetic sensitive layer films into powder and then reprint them on the flexible substrate [71]. The surface wrinkling is the method described in the previous article to obtain wrinkles after thermal or mechanical induction of pre-strain of elastic film. The transferring method is to deposit the rigid multilayer film on the Si substrate and then transfer the film to the flexible substrate with folded structure by transfer printing. The rigid islands are to prepare a substrate with a modulus gradient. The area with low modulus is soft as the elastic stretch area; the area with high modulus is hard as rigid islands. The elastic region with low modulus can bear large deformation and keep the rigid island region with high modulus where the functional equipment is located in an ideal strain-free state, avoiding the crack generation of GMR multilayer film. The printing method is to use the adhesive solution to re-contact the magnetic-sensitive thin film, which can realize the micro-structure processing of the pattern, and the stretchability of the device will depend on the flexible substrate rather than the GMR multilayer film.

### 2.4. Flexible TMR Sensor

In 1975, the tunnel magnetoresistance (TMR) effect was discovered by Julliere [73] in the magnetic tunnel junction (MTJ), which is the effect of tunnel resistance changing with the relative direction of ferromagnetic material, appearing mostly in free layer/barrier layer/reference layer structure. The TMR ratio can be expressed as

(4) $$TMR=\frac{2p_{1}p_{2}}{1-p_{1}p_{2}}$$

where $p_{1}$ and $p_{2}$ are spin polarizability of the reference layer and free layer, respectively.

**Figure 9:**
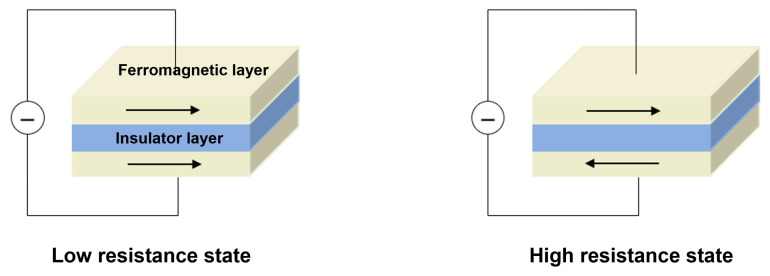
Working principles of TMR sensor.

The magnetization direction of reference layer remains constant under a certain magnetic field, while the magnetization direction of free layer can rotate under the action of external magnetic field. As shown in Figure 9, the resistance of the tunnel junction changes with the angle of the relative magnetization direction between the free layer and the reference layer: when the magnetization directions of the free layer and the reference layer are antiparallel, the resistance value is the maximum, while when the magnetization directions are parallel, the resistance value is the minimum [74,75].

The linear region where the resistance varies with the magnetic field is the working interval of the sensor. Different magnetization configurations in the reference layer and free layer can achieve linear hysteresis. Several methods have been proposed to reduce the hysteresis in magnetoresistance sensors and show a linear response, for example, varying the thickness of the sensing layer.

Compared with GMR sensors and AMR sensors, TMR sensors have higher magnetic field sensitivity and possess several advantages, including small size, high response frequency, and high sensitivity [76]. They are widely used in applications of reading head [77], non-destructive testing (NDT) [78,79,80,81,82], current detection, magnetic field imaging [83,84], and encephalography techniques [85].

With the development of flexible GMR sensors, TMR sensors are also gradually endowed with flexible characteristics [86]. However, developing spintronic devices on flexible organic substrates without sacrificing performance, that is, maintaining the original electrical characteristics when the sensor is bent remains challenging. First, the surface roughness of most flexible substrates is worse than that of rigid silicon wafers, which degrades the performance of magnetic films grown on flexible substrates. Second, micromachining can also lead to degradation of the TMR sensor because of the limited spatial resolution and the damage to the polymer substrate and its magnetic film during heating, physical or chemical etching processes. Finally, the low glass transition or melting temperature of most polymer films makes it difficult to withstand annealing processes that require high temperatures above 300 °C, which is necessary for MTJ deposition by sputtering. To overcome these difficulties, Barraud et al. successfully deposited large area MTJ structures directly on a flexible transparent organic substrate by sputtering a test device using a shadow mask technique [86], opening the way to the development of MTJ-based spintronics. Subsequently, the possibility of integrating tunnel junction elements into bendable membranes was demonstrated [87], and studies [88] have been reported on the fabrication of TMR sensors using a transfer printing process where MTJ structures on rigid substrates are transferred to flexible substrates to avoid difficulties in annealing temperature.

Recently, Kapton was proved to be a very promising flexible substrate for magneto-electronic applications, fully compatible with the integration of spintronic devices with fine interface layer characteristics as well as advanced manufacturing technologies such as electron beam lithography. Therefore, the Al_2_O_3_-based MTJ could be sputtered directly on the Kapton substrate, and the TMR ratio remained at 12% at room temperature during bending. In addition, it can be further imprinted directly on it by electron beam lithography to obtain microscopic patterns that are comparable in quality and resolution to conventional rigid substrates [89]. Then, Gaspar et al. reported an integration of MTJ sensing equipment on a flexible substrate with a reluctance response of over 150% and a sensitivity change of 7.5% when the bend radius was reduced to 5 mm [90].

**Figure 10:**
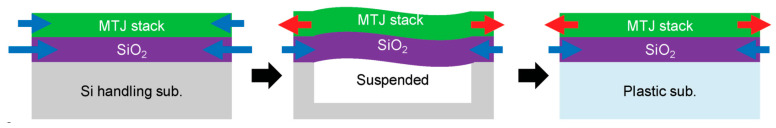
Schematic diagrams of transferring the MTJ stack. Adapted with permission from Ref. [91]. Copyright 2016, copyright John Wiley & Sons, Inc.

By using single crystal MgO instead of amorphous Al_2_O_3_ as the tunneling layer, the TMR ratio can be improved by several orders of magnitude because MgO lattice symmetry has a screening effect on the wave function of tunneling electrons, thus making an additional contribution to TMR. Therefore, they are promising candidates for flexible electronic applications. In order not to compromise the specific crystal structure of MgO barrier MTJ during manufacturing, Loong et al. demonstrated a flexible MgO barrier layer MTJ device manufactured using a transfer printing process that demonstrated reliable and stable operation in the presence of substantial deformation of the device substrate [91]. In their approach, patterned tape layers are applied to protect the MTJ device area, and then the sacrificial silicon is etched away by isotropic etching (Figure 10). The improved performance of the flexible MgO barrier MTJ in the pseudo-spin valve is obtained by means of the strain relaxation of SiO_2_ layer.

**Figure 11:**
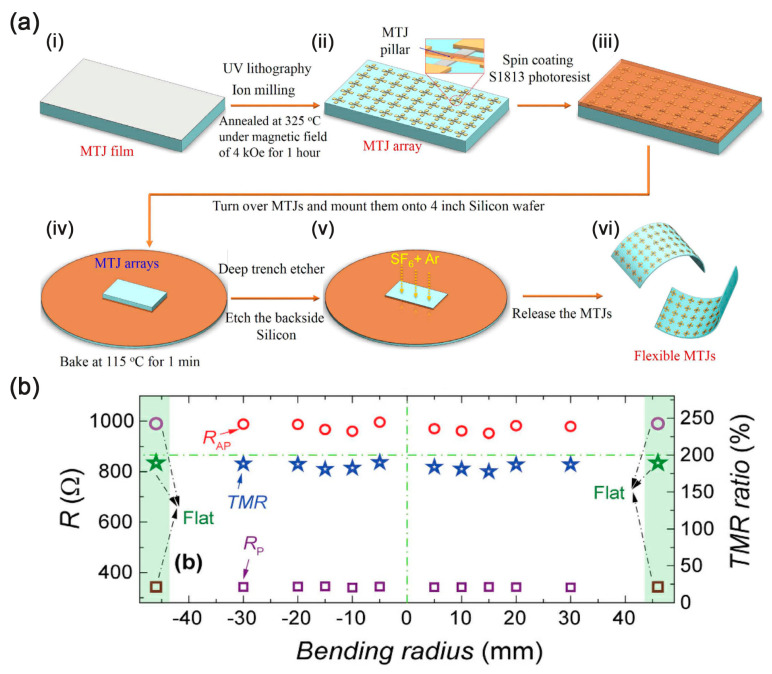
(**a**) Schematics on the fabrication process of flexible TMR sensors; (i) MgO-barrier MTJ stacks on thin thermally oxidized silicon wafer; (ii) MgO-barrier MTJ array after patterning; (iii) A layer of S1813 photoresist spin-coated onto the device surface; (iv) The sample was turned over, mounted onto a four-inch silicon wafer covered with photoresist; (v) Thin the back side of the silicon; (vi) Flexible MgO-barrier MTJs after removing the photoresist; (**b**) Resistance in the parallel (R_P_) and antiparallel (R_AP_) states and TMR ratios varying with bending radius. Adapted with permission from Ref. [92] and licensed under CC BY 4.0. Copyright 2017, copyright Springer Nature Limited.

For freestanding silicon films, further bending to smaller radii of curvature becomes risky as the tiny shear strain causes it to break. To further improve the bending capability of the device, Chen et al. grew high-performance MgO-barrier MTJs directly onto ultrathin flexible silicon membrane with a thickness of 14 μm and then transfer-and-bond to plastic substrates like Kapton (Figure 11). This flexible MTJ was fully functional and exhibited a TMR ratio of up to 190% at bending radii as small as 5 mm with excellent stability [92]. Selma et al. improved by using a (001) orientated MgO barrier, achieving a TMR ratio of over 150% at room temperature. MTJ stacks are manufactured on a silicon substrate, etching the backing of the substrate using deep reactive ion etching (DRIE) to obtain MTJ in a flexible silicon substrate, avoiding unnecessary heating of the equipment and allowing wireless operation [93]. These flexible MgO barrier MTJs open a path to high-performance spintronic devices in flexible and wearable device applications.

### 2.5. Flexible MI Sensor

**Figure 12:**
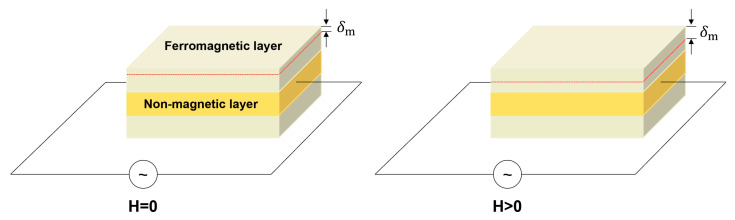
Working principles of MI sensor.

The magnetoimpedance (MI) effect refers to the change of electrical impedance of soft ferromagnetic material when applying a magnetic field. As shown in Figure 12, this effect is basically the result of a reduction in the effective cross section of the material when the alternating current (AC) flows due to the skin effect. It is similar to large changes in the real and imaginary parts of the sensor’s complex impedance, controlled by changes in the material’s permeability caused by an applied magnetic field, driven by AC when exposed to a magnetic field [94]. The output impedance (𝑍) can be calculated by resistance (𝑅) and reactance (𝑋) that is Z=R+jX=R+jωL , where ω is driven angular frequency of alternating current, and 𝐿 is GMI sensor inductance. According to Maxwell’s equation, the impedance of a planar magnetic rectangular thin strip can be written as [95]

(5) $$Z=R_{dc}\cdot jka\cdot coth\;(jka)$$

where $R_{dc}$ is the dc resistance of conductors, 2 a; the thickness, and $k=(1+j)/\delta_{m}$ , with imaginary unit j . $\delta_{m}$ is the penetration depth of magnetic medium [95],

(6) $$\delta_{m}=\frac{c}{\sqrt{4\pi^{2}f\sigma \mu_{T}}}$$

where c is the speed of light, σ the electrical conductivity, $\mu_{T}$ the transverse permeability, and f=ω/2π is the frequency of the ac flowing along the sample.

Because of the discovery of the new effect in magnetically coated non-magnetic microwires, the MI sensor based on this effect can operate at room temperature, has demonstrated extraordinary sensitivity, and has been implemented in devices used in the automotive, aerospace, and medical fields. The cost effectiveness and high ubiquity of MI devices indicate a wide range of applications in biology and medicine, for example, non-invasive monitoring of biological magnetic fields [85,96].

So far, the work on flexible MI sensors is very limited. Fernandez et al. obtained the first nanostructured multilayers with excellent GMI response on a flexible substrate, which is a F/C/F (F is a ferromagnetic layer, and C is a conductive nonmagnetic layer) sandwich structure created by inserting conductive nonmagnetic layer between two ferromagnetic layers onto a flexible polymer substrate. This sandwich enhances the magnetic induction effect and allows for a higher GMI ratio when the conductivity difference between the two layers is sufficiently large, with an MI ratio of 110% and a sensitivity of 2.2 × 10^5^%/T operating at 150 MHz. In addition, the response of samples deposited on flexible substrates to applied pressure was studied, and the GMI curve varied greatly as a function of pressure [94]. 

**Figure 13:**
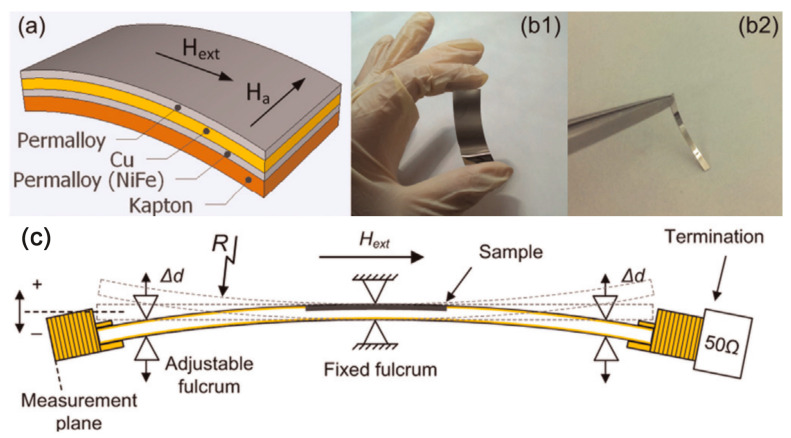
(**a**) The device architecture of the flexible GMI sensor based on a Kapton substrate; (**b1**,**b2**) Physical picture of the flexible GMI sensor; (**c**) Schematic diagram of deflection measuring device for flexible GMI sensor. Adapted with permission from Ref. [97]. Copyright 2014, copyright Elsevier B.V.

Excellent MI applications require soft magnetic materials with low coercivity, high permeability, high saturation magnetization, and well-defined magnetic anisotropy, and F/C/F sandwich structures can significantly enhance the MI effect by concentrating the current within the conducting layer. With the demonstrated potential of Kapton in flexible electronics, a flexible MI sensor has been prepared using NiFe/Cu/NiFe three-layer material on a Kapton substrate (Figure 13). Deflection measurements of the MI sensor have been performed for the first time on a flexible microstrip transmission line in the wide frequency range of 0.1 to 3 GHz. The three-layer sensor structure was prepared by electron beam evaporation on a flexible substrate, and a constant magnetic field is applied along the short axis of the PI film substrate to induce transverse anisotropy in the magnetic layer. Magnetic field response and frequency response of MI sensor and their dependence on sensor deflection were studied by using customized flexible microstrip transmission line. It can be used for motion or position detection in wireless applications in wearable or robotic technology [97].

Agra et al. reported MI effect results over a wide frequency range for non-magnetostrictive multilayer Py/(Ag, Ta) films grown on glass and flexible substrates and concluded that the results were very similar regardless of the substrate used, and good MI responses were obtained [98]. For flexible MI devices, designing effective combinations of films/polymers with complementary properties is required. Cyclo olefine polymer films have high transparency (92% transmittance) and good turbidity and enhanced brightness, which are good candidates for “dual” designs, including optical and magnetic detection [99]. Kurlyandskaya et al. deposited GMI components on rigid and flexible substrates at different deposition rates using sputtering technology, compared and analyzed their structure, magnetic properties, and MI, and found that the sensor performance on glass substrate could be basically reproduced on cyclic olefin polymer substrate [100].

**Figure 14:**
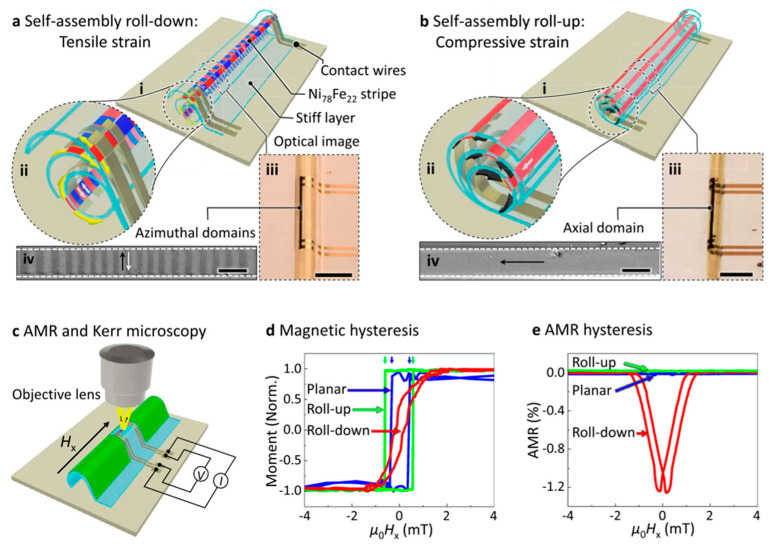
Magnetic properties of flexible GMI sensors induced by strain by self-assembly rolling approach. Adapted with permission from Ref. [101] and licensed under CC BY 4.0. Copyright 2022, copyright Springer Nature Limited.

Because of the planar configuration of the magnetic layer, it is still necessary to define transverse to spool magnetization by annealing the planar structure at temperatures in excess of 200 °C and under an applied current of at least 30 mA. This process is difficult to implement on a single sensor integrated circuit and even more difficult for array configurations. Karnaushenko et al. proposed a platform that allows stimulus control to self-assemble initial flat NiFe/Cu/NiFe heterostructures to a 3D tubular structure with GMI capabilities, resulting in circular magnetization and avoiding high-temperature processing where current is applied. They integrated a variety of functional components, including pick-up coils and GMI sensors, into a tubular structure that is typically 2 mm in length and approximately tens of microns in diameter. When converted to a 3D architecture, their magnetoelectric performance was dramatically improved by a factor of 80. This improvement is due to geometrically induced circumferential magnetization achieved when transforming the initial planar sensor into a tubular structure. That is, extending a 2D ferromagnetic structure to a 3D curved geometry can adjust its magnetism [96]. Then Singh et al. obtained a platform by applying a polymer platform to a self-assembly rolling technique that allows uniform and controlled bending of the functional layers adhering to it, regardless of its shape and size (Figure 14). The specially designed self-assembled “Swiss coil” architecture allows the geometry of the winding film to be adjusted, thereby adjusting the strain and the anisotropy of any magnetic micron-scale geometry prepared on its surface, addressing the effect of curvature on the magnetization caused by the applied strain [101].

With the flexibility of MI sensors, in the work of [102], magnetic sensing components and permanent magnet nanocomposite artificial cilia integrated on highly elastic films have been reported as a new type of tactile sensor. The nanocomposite is made of iron nanowire (NW) mixed with PDMS. Cilia utilizes the NW’s permanent magnet behavior, allowing remote operation without the need for additional magnetic fields to magnetize the NW. The sensor works by detecting changes in the ciliary magnetic field, which is generated when iron NW is deflected by external forces, and a GMI sensor located below the cilia measures changes in the magnetic field. Thus, in the presence of external forces, the cilia bends, causing a change in the average magnetic field value detected by the MI sensor and a change in its impedance.

### 2.6. Flexible ME Sensor

**Figure 15:**
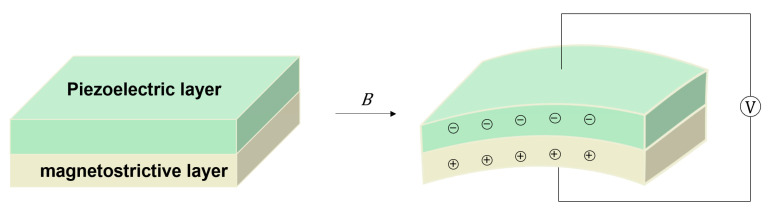
Working principles of ME sensor.

The magnetoelectric (ME) effect refers to the coupling effect between magnetization and electric polarization, that is, the induced dielectric polarization of a material under the action of an applied magnetic field or the induced magnetization under the action of an applied electric field. It was first observed in Cr_2_O_3_ in 1961 [103,104]. Due to the weak ME effect of single-phase materials, as shown in Figure 15, a composite composed of piezoelectric and magnetostrictive materials was designed to study the magnetoelectric effect caused by strain transfer between ferromagnetic and ferroelectric phases [105,106,107]. 

With the advancement of modern science and technology, the application of magnetoelectric effect in magnetic field sensor has become a research hotspot in many fields such as medical treatment, industry, and biology because of its advantages of small portability, high sensitivity, simple structure, and low cost [108].

The fabrication method of ME composite has always been the focus of researchers. To overcome the rigid and brittle problem and open up new applications, the flexible and brittle ME composite materials based on piezoelectric polymers, such as nano-composite materials, polymers as binder, and laminate composite materials, have been studied [109,110,111]. Poly (vinylidene fluoride) (PVDF) and its copolymer show great potential due to its high piezoelectric coefficient, good stability, low loss, low processing temperature, and good flexibility, and attract more and more attention. Due to the magnetic driving characteristics, a representative magnetoelectric composite can be obtained by adding magnetoelectric nanoparticles to the PVDF matrix. Because PVDF can improve the magnetic properties of composite structures, it has been the focus of researchers [106,108,111,112,113].

In addition, the preparation of other magnetoelectric nanocomposites is also worthy of further study. Verma et al. obtained multiferroic 0.25BaTiO_3_–0.75CoFe_2_O_4_ nanocomposites by hydrothermal synthesis, which achieved strain-mediated ME effect and could effectively regulate magnetic anisotropy [114]. A large lattice mismatch (>10%) system consisting of epitaxial BaTiO_3_ matrix and Ni nanocrystals was designed, which exhibits dielectric properties with extreme sensitivity to be applied to weak magnetic fields [115]. Additive manufacturing techniques [116] can also introduce magnetic particles into polymer composites to process and integrate ME nanocomposites such as 3D printing [117,118], screen printing [119], and spray-printing [120].

Aiming at the influence of magnetic field direction and composite material thickness on magnetic field and ME response, Martins et al. investigated the fabrication of ME composites using a simplified solvent casting method using piezoelectric phase PVDF-TrFE and magnetostrictive phase CoFe_2_O_4_ (CFO) nanoparticles without vacuum treatment. They found that the presence of low content nanoparticles in the composites significantly improves the polarization and piezoelectric response of the copolymer matrix, which is a promising candidate for piezoelectric and ferroelectric applications at room temperature [121].

**Figure 16:**
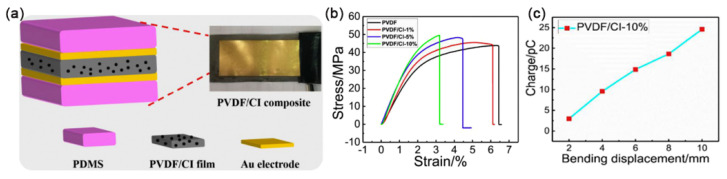
(**a**) Schematics of PVDF/CI composite structure; (**b**)Tensile stress-strain curves of PVDF/CI films of CI particles in different content; (**c**) Piezoelectric charge variation of PVDF/CI-10% films at different bending displacement. Adapted with permission from Ref. [112]. Copyright 2018, copyright Elsevier B.V.

Magnetic particles used in magnetic PVDF polymer-based composites have the requirement of high saturation magnetization. Carbonyl iron (CI) particles are metallic magnetic powders with soft magnetic properties. Magnetic CI particles have the advantages of high saturation magnetization, high permeability, and good thermal stability, so they are a good choice for magnetic PVDF polymer matrix composites. As shown in Figure 16, Sang et al. prepared a multifunctional polyvinylidene fluoride/carbonyl iron (PVDF/CI) composite film by doping magnetic CI particles into PVDF matrix by solution casting process. Experiments show that CI particles increase Young’s modulus and maximum tensile strength of the composite film, PVDF/CI film is sensitive to deformation, and the piezoelectric charge signal depends on the bending displacement. The larger the deformation, the more piezoelectric charge, which shows positive piezoelectric effect [112].

The dispersion and magnetic response characteristics of magnetic nanoparticles are very important for the sensitivity and stability of flexible magnetic field sensors. To improve the performance of magnetic nanoparticles, Song et al. reported a preparation method of fullerene-coated ferric oxide (C_60_@Fe_3_O_4_) magnetic nanoparticles. Among them, the high elastic rubber styrene ethylene butylene styrene (SEBS) film substrate was prepared by direct forming method, which can adhere to sensitive nanomaterials, increase the deformation of the film with magnetic field, and fix carbon nanotubes (CNTs) with excellent electrical properties and magnetic nanoparticles as sensitive units, to prepare flexible magnetic field sensors. It shows good magnetic response performance [108].

To further increase the ME coefficient, composite materials usually require a permanent magnet or electromagnet to provide a biased magnetic field, which not only makes the sensor bulky but also brings large electromagnetic noise. Polymer-based multi-iron composite with self-biased ME effect is expected to solve this problem. Jing et al. reported a self-biased polymer matrix ME nanocomposite (M/P/M). They used CFO nanoparticles and multi-walled CNTs as a PVDF matrix of polyvinylidene, with a poly (vinylidene fluoride-trifluo-roethylene) (P (VDF-TrFE)) layer (P layer) sandwiched between two CFO-CNT-PVDF conducting layers (M layer). Uniaxial tensile test was carried out to reveal the effect of CFO content on mechanical properties of composites [106]. Then, large self-biased 0–3 type P(VDF-TrFE)/CoFe_2_O_4_ flexible ME composite films were proposed using solution casting method by Mu et al. The results showed that the nano-particles are uniformly dispersed in the P(VDF-TrFE) matrix, and the composite films have excellent ME effect [107].

For ME laminates, the limitation is the low permeability of the magnetostrictive layer. To solve this problem, Zhai et al. prepared a novel ME laminate made from a small-strain but high-permeability Metglas layer laminated with PVDF. This Metglas/PVDF single crystal and triple sandwich configuration of ME laminates are very thin, flexible, and inexpensive, with a large ME voltage coefficient and excellent sensitivity to small changes in AC and direct current (DC) magnetic fields [105].

Reis et al. prepared Fe_61.6_Co_16.4_Si_10.8_B_11.2_ (FCSB)/PVDF/FCSB ME laminates using PVDF with chemical and thermal stability, flexibility, and high resistivity, and FCSBS with high permeability, high pressure magnetic coefficient, and magnetic anisotropy, and they fabricated an innovative magnetic field sensor based on the laminates. Based on the anisotropic ME effect, this sensor can detect the magnitude and direction of AC and DC magnetic fields, which is comparable to other traditional types of sensing technology, such as GMI and GMR [122]. In addition, the charge change signal generated by ME composite through piezoelectric layer is easily affected by electromagnetic interference and noise. Reis et al. chose to use a charge amplifier circuit to optimize ME signal processing conditions and improve circuit configuration because it converts the charge signal into a voltage signal, which amplifies the initial amplitude and filters out low frequency noise. By integrating a charge amplifier, an AC-RMS converter, and a microcontroller with an on-chip ADC, the development of a DC magnetic field sensor based on PVDF/Metglas composites were reported, and the ME voltage response was undistorted, remaining linear [109].

In recent years, the printing process has been used to develop flexible electronic devices and sensors that are cost-effective and lightweight. Chlaihawi et al. prepared a novel ME thin film sensor on a flexible and magnetic Metglas substrate by silk-printing PVDF as a piezoelectric layer, which can be used for the detection of AC magnetic field at room temperature [110].

**Figure 17:**
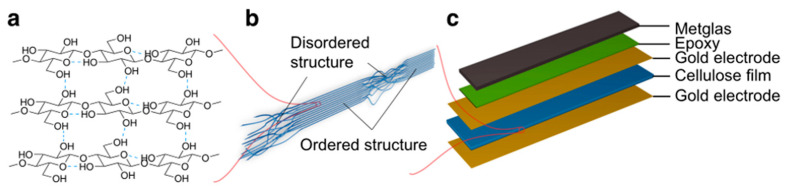
Schematics of the cellulose based flexible ME sensors. (**a**) Scheme of cellulose crystal II, the most common crystalline type in regenerated cellulose materials; (**b**) Illustration of cellulose fibril alignment at the cross-section of cellulose film; (**c**) cellulose based ME laminate structure. Adapted with permission from Ref. [123] and licensed under CC BY 4.0. Copyright 2017, copyright Springer Nature Limited.

When an external magnetic field is applied to the ME composite, the magnetic element changes its shape, thereby causing strain on the piezoelectric element and achieving dielectric polarization. This unique indirect two-corresponding variable coupling offers the possibility of optimizing the flexibility of the piezoelectric phase, magnetostrictive phase, and their interface to enhance ME response. Zong et al. demonstrated the fabrication of ME composites based on cellulose, a natural biopolymer, containing a laminated structure of Metglas and cellulose films (Figure 17). This achieved two corresponding variable couplings to enhance the ME response, which is superior to the composite structure reported previously [123].

In inorganic flexible ME sensors, ME heterogeneous laminates show low ME coefficient and low mechanical compliance. Yang et al. prepared a highly flexible heterostructure ME sensor composed of Metglas foil and an all-inorganic flexible piezoelectric PZT thick film on a two-dimensional mica substrate, which obtains a large ME coefficient of 19.3 V/cm·Oe and ultra-high sensitivity of 200 nT at low frequency, and showed excellent mechanical fatigue performance after 5000 bending cycles [111].

## 3. Applications of Flexible Magnetic Sensors

Flexible magnetic sensors have gained increasing interest in the fields of wearable and implantable technology, flexible electronics owing to their flexibility, sensitivity, magnetic stability, biocompatibility, and easy production and preservation.

### 3.1. Geomagnetosensitive E-Skins

**Figure 18:**
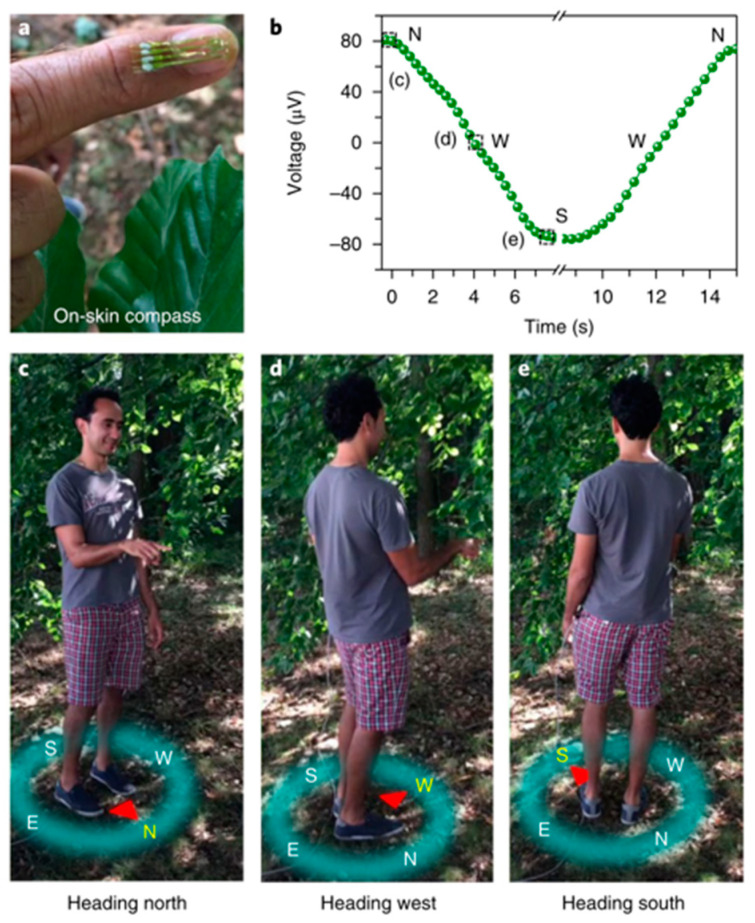
Outdoor geomagnetic exploration by E-skin compass. (**a**) E-skin compass attached to the finger; (**b**) Time evolution of the output voltage of the e-skin compass when the person rotates back and forth from the magnetic north (N) to magnetic south (S) via west (W); (**c**–**e**) Pictures of people facing N, W, and S. Adapted with permission from Ref. [31]. Copyright 2018, copyright Springer Nature Limited.

Objects with geomagnetic induction can orient themselves by sensing the Earth’s magnetic field for navigation. Magnetic sensors have been proven to have the potential to interact with objects in a non-contact manner. It needs to operate when only the geomagnetic field is present without any external magnetic bias interference, which eliminates the need to install permanent magnets to properly alter the Earth’s magnetic field, thus simplifying the ability to implement artificial magnetic receiving devices on human skins. The ensuing challenge is that the device needs to be spatially oriented in a geomagnetic field of only 50 μT. A highly compliant E-skin compass based on an AMR sensor allows a person to orient with respect to the Earth’s magnetic field [31]. As shown in Figure 18, the AMR magnetic field sensor is manufactured in polymer foils of 6 μm thick and can be perfectly attached to the finger to detect geomagnetic fields (40–60 μT) with no loss of function even at a bend radius of 150 μm. Combining with a game engine, the device could create a virtual reality environment driven by the movement of the hand in the earth’s magnetic field. Combined with the human body, the device could be oriented outdoors through the Earth’s magnetic field. With high sensitivity, excellent flexibility, and stability, the compass provides a platform for human positioning in outdoor environments and contactless interaction in virtual reality.

### 3.2. Magnetoelectric Compass

Navigation applications require sensors that indicate the direction and orientation of DC (or AC) magnetic field. ME sensors with strain-mediated ME structures respond only to magnetic fields along a specific direction but are insensitive to the angle of a randomly oriented in-plane AC magnetic field. Wu et al. proposed an electromagnetic compass that can detect the intensity and direction of the in-plane AC magnetic field, as well as a magnetic sensor that can measure the amplitude and direction of the in-plane AC magnetic field [124]. The barbell structure of the compass makes the NdFeB permanent magnet produce compressive stress on the piezoelectric element through torque effect. In the case of no DC bias magnetic field, magnetic electrocoupling effect is mediated by stress. The device has good detection performance for the in-plane AC magnetic field in any direction, its strength sensitivity is 0.01 Oe, and angle sensitivity is ±0.2°. In addition, the barbell structure has the advantages of simple structure, stable performance, and durability, and has broad application prospects in angle sensor and compass.

### 3.3. Non-Contact Interactive Platform

**Figure 19:**
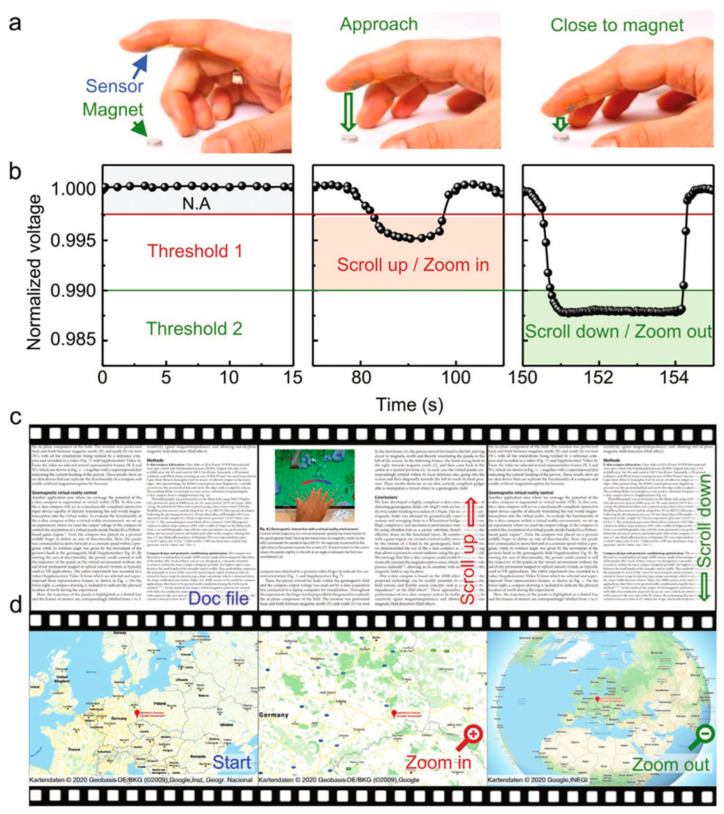
Highly flexible printed GMR sensors for Non-contact Interactive Platform. (**a**) A compliant printed GMR sensor attached to the finger which can read displacement of a permanent magnet; (**b**) The time evolution of the normalized sensor read out dependent on the distance between the finger and the magnet; The sensor signal is used to navigate through the document (**c**) or zoom in/out the map (**d**). Adapted with permission from Ref. [72] and licensed under CC BY 4.0. Copyright 2021, copyright John Wiley & Sons, Inc.

Unlike the thermal, tactile, and chemical sensors commonly used in traditional electronic products, flexible magnetic field sensors have the property of remote action and can rely on the surrounding magnetic field to achieve non-contact skin interaction. They are characterized by compact, no light requirements, and can be driven by ubiquitous magnetic fields, facilitating the realization of magnetically sensitive E-skins for interactive devices. As shown in Figure 19, a printed flexible GMR sensor was fabricated by dispersing [Py/Cu]_30_ GMR microflakes in a Styrene-butadiene-styrene (SBS) elastomer, which is able to perform detection at low magnetic fields below 1 mT and maintain high performance reluctance sensing at extreme mechanical deformation of up to 16 µm bending radius and 100% stretch [72]. This printed GMR sensors can be easily applied to the skin to enable wearable interactive electronics suitable for everyday use by the public, allowing non-touch control of virtual objects, including zooming in and out of interactive maps, and scrolling through electronic texts.

**Figure 20:**
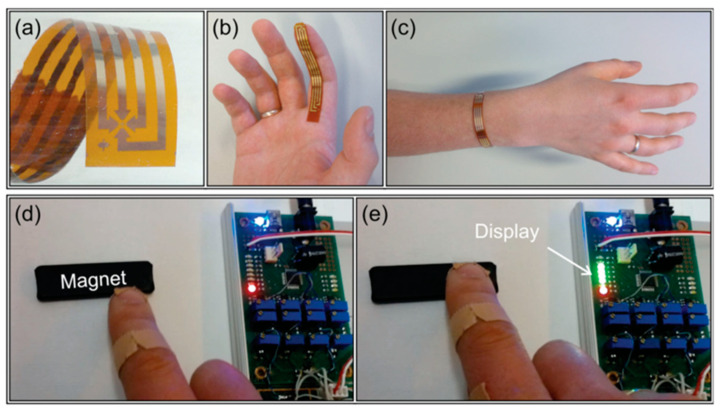
Flexible Hall sensor attached to the human body for interactive pointing devices. (**a**) Magnified view of the flexible Hall sensor; (**b**,**c**) Flexible Hall sensor attached to the human body; (**d**,**e**) The relative position of the finger with respect to a permanent magnet is displayed in real time by monitoring the sensor output. Adapted with permission from Ref. [18]. Copyright 2015, copyright John Wiley & Sons, Inc.

This capability is not limited to the GMR sensor, which enables non-contact interaction based on any of the magnetic field sensing principles. There are also studies that rely on the Hall effect to produce highly flexible magnetic field sensors that exhibit very good mechanical, thermal, and chemical stability and can be applied to the most advanced consumer electronics [18]. The resulting sensor can be attached to a finger to create an interactive pointing device and build a wearable electronics component that detects the permanent magnet’s position and outputs it (Figure 20).

### 3.4. Hybrid Rigid-Flexible Probe

The development of TMR sensors provides a lower cost system for magnetic encoder-based motion systems that can withstand harsher industrial environments. Their spatial resolution enhances the detection of microruler rod spacing, improves working distance and tolerances imposed by industry. However, sensor packages designed to protect equipment from mechanical damage result in lower sensor performance and micromachining yield due to their rough underlying layer. Franco et al. successfully implemented a magnetic encoder based on TMR sensors for positioning and motion systems in harsh environments, combining state-of-the-art TMR sensors with packaging schemes that have minimal impact on performance [77]. At its core, there is the stiff-flexible hybrid MR Probe, which consists of combining a sensing chip with a flexible PI connector. The flexible PI connector establishes electrical contacts away from the Si sensing chip only by pushing the contacts to the back of the chip or to a lower level parallel to the sensor plane to achieve a minimum distance of 7.5 μm between the sensor and the magnetic source.

### 3.5. Intelligent Medical Detection

The treatment or diagnosis of the cardiovascular system requires the insertion of a cardiac catheter, which is usually followed up with X-ray imaging and contrast media. It takes several attempts to make sure the catheter is in the correct position and orientation in the heart, which leads to the dangerous overuse of contrast media and X-ray doses. To avoid these problems, the flexible TMR sensor can be attached to the catheter tip with a 100-fold reduction in thickness and weight. Selma et al. fixed a flexible TMR sensor with high performance, flexibility, and mechanical durability on the tip of a 2 mm diameter cardiac catheter, applied a magnetic field ranging from −50 to 50 Oe with Helmholtz coil, and found that the sensor still maintained its performance and showed significant response to magnetic fields at different angles [93]. Therefore, it can be used to develop highly miniaturized catheter systems for directional tracking with minimal side effects and can be further used as a part of directional monitoring systems in miniaturized instruments with embedded electronics.

## 4. Summary and Future Perspectives

In conclusion, we provide an overview of recent achievements in the field of flexible magnetic sensors. Some of the most important features for these flexible magnetic sensors are presented in Table 1. In recent years, based on new sensitive materials, new sensing structures, and new fabrication techniques, flexible magnetic sensors already have numerous exciting demonstrations in the fields of magnetic sensitivity, stability, and tensile properties. However, they are still in the initial stage of technology, and compared with other flexible and elastic sensor parts, stability, tensile ability is still a little insufficient.

It is inevitable that the magnetic sensing performance of flexible magnetic sensor under stress is different from that without stress as the magnetism of magnetic material is very sensitive to stress. Therefore, structural design of magnetic thin films is an effective way to regulate their stress-sensitive properties; however, compatibility of these methods with the microfabrication of devices is a challenge.

Although the tensile amount of the prepared flexible magnetic films in one direction has exceeded 200%, these films basically do not have good multi-direction tensile ability, and its long-term stability after deformation of more than 1000 loading cycles must be ensured, to meet the application requirements in the wearable field. The development of flexible magnetic films with excellent multidirectional stretching ability is an important area that warrant exploration.

Although the above application techniques are demonstrated based on a single sensing principle, they can be easily extended to other magnetic functional and even non-magnetic components. For example, based on anisotropic reluctance effects or planar Hall effects, printable and flexible high-performance magnetic field sensors can be achieved by using printing technology to apply viscous triblock copolymers. All the flexible magnetic field sensors that are sensitive to the in-plane component of the magnetic field can realize 2D or even 3D positioning and navigation in harsh environments. Flexible magnetic sensors that use highly compliant copolymers as substrates or packages can uniformly cover highly non-planar surfaces, preventing long-term damage from aggressive body fluids, and have the characteristic of biocompatibility for practical use on the skin and in the body.

In the future, the flexible magnetic sensors with high sensitivity, high stability, and high flexibility will be used as a navigation detection part in soft robots, medical implants, smart skin, virtual reality, and augmented reality interactive devices. By attaching multiple flexible magnetic sensors or arrays to the surface of the robot hand or other machine parts, the motion state and position information of multiple positions can be obtained at the same time to achieve accurate driving. Highly sensitive flexible magnetic sensors will enable real-time monitoring of muscles, joints, and heart valves with magnetic sensing capabilities, advancing health monitoring, diagnosis, and environmental sensing to a new realm of possibilities. They can provide magnetic functions for smart textiles, which can be applied to contactless interactive devices that will bring exciting possibilities for business, gaming, health monitoring, and fitness training. With the rapid development of machine learning and artificial intelligence, they will be an important development direction of flexible magnetism in the future to focus on the electrical and mechanical interfaces with other soft electronic components. This will involve integrating flexible magnetic sensors with other functional component platforms to construct artificial intelligence network systems with various sensing and response functions.

## Figures and Tables

**Table 1:** Comparison of technologies for flexible magnetic sensors. The table summarizes some sensor’s features such as materials, substrate, thickness, sensitivity, technique, minimum bending radius, and maximum tensile strain.

Type	Material	Substrate (Thickness)	Sensitivity	Technique	Minimum Bending Radius/ Maximum Tensile Strain	Ref.
Hall	graphene	Kapton (50 µm)	79 V/AT	chemical vapor deposition	4 mm	[17]
	Bismuth	Kapton (100 μm)	−2.3 V/AT	magnetron sputtering	6 mm	[18]
	4 chips	Kapton (150 μm)	59 ± 1 V/AT	CMOS	10 mm	[22]
AMR	Py	PET (6 μm)	5.4 V/T	e-beam evaporation	0.15 mm	[31]
	Py	PET (100 μm)	5 V/T	magnetron sputtering	5 mm	[28]
GMR	Co/Cu multilayers	PDMS (40 µm)	-	magnetron sputtering	4.5%	[60]
	Co/Cu and Py/Cu multilayers	PET (1.4 μm)	220%/T	pre-strain induced mechanically	0.003 mm/270%	[66]
	Py/Cu multilayers	SBS (3 μm)	300%/T	screen printing	0.016 mm/100%	[72]
	Py/Cu multilayers	PDMS (60 µm)	220%/T	pre-strain induced thermally	30%	[65]
TMR	Co/Al_2_O_3_-based	Gel-film (300 µm)	-	DC sputter	15 mm/1%	[86]
	MgO-barrier	Si (3–5 μm)	49,300%/T	deep-reactive-ion-etching	0.5 mm	[93]
MI	Py/Cu/Py	imide- and acrylic-based polymers	500,000%/T	CMOS	-	[94]
	Py/Cu/Py	Kapton (125 µm)	92,000%/T	e-beam evaporation	72 mm	[97]
ME	(P(VDF-TrFE)/CFO	-	-	solution casting	-	[107]
	FCSB/PVDF/FCSB	-	150 V/T 14,000 V/T	single-roller quenching	-	[122]

## Data Availability

Not applicable.
