# Correction: Pan et al. Flexible Magnetic Sensors. *Sensors* 2023, *23*, 4083

**DOI:** 10.3390/s25092838

**Published:** 2025-04-30

**Authors:** Lili Pan, Yali Xie, Huali Yang, Mengchao Li, Xilai Bao, Jie Shang, Run-Wei Li

**Affiliations:** 1 CAS Key Laboratory of Magnetic Materials and Devices, Ningbo Institute of Materials Technology and Engineering, Chinese Academy of Sciences, Ningbo 315201, China; 2 Zhejiang Province Key Laboratory of Magnetic Materials and Application Technology, Ningbo Institute of Materials Technology and Engineering, Chinese Academy of Sciences, Ningbo 315201, China; 3 School of Future Technology, University of Chinese Academy of Sciences, Beijing 100049, China

In the published publication [1], there was an error regarding the affiliations for Lili Pan. The updated affiliations should include the following:

1. CAS Key Laboratory of Magnetic Materials and Devices, Ningbo Institute of Materials Technology and Engineering, Chinese Academy of Sciences, Ningbo 315201, China
2. Zhejiang Province Key Laboratory of Magnetic Materials and Application Technology, Ningbo Institute of Materials Technology and Engineering, Chinese Academy of Sciences, Ningbo 315201, China
3. School of Future Technology, University of Chinese Academy of Sciences, Beijing 100049, China

The authors state that the scientific conclusions are unaffected. This correction was approved by the Academic Editor. The original publication has also been updated.
